# Study design and baseline characteristics of a combined educational and environmental intervention trial to lower sodium intake in Swiss employees

**DOI:** 10.1186/s12889-018-5366-0

**Published:** 2018-04-02

**Authors:** Sigrid Beer-Borst, Xhyljeta Luta, Stefanie Hayoz, Kathrin Sommerhalder, Corinna Gréa Krause, Julia Eisenblätter, Sandra Jent, Stefan Siegenthaler, Rafael Aubert, Max Haldimann, Pasquale Strazzullo

**Affiliations:** 1 Institute of Social and Preventive Medicine, University of Bern, Finkenhubelweg 11, 3012 Bern, Switzerland; 2 Department of Health Professions, Bern University of Applied Sciences, Murtenstrasse 10, 3008 Bern, Switzerland; 3 Federal Food Safety and Veterinary Office, Division of Risk Assessment, Laboratories, Schwarzenburgstrasse 155, 3003 Bern, Switzerland; 4 Department of Clinical Medicine & Surgery, Federico II University of Naples Medical School, via S. Pansini 5, 80131 Naples, Italy

**Keywords:** Workplace health promotion trial, Educational intervention, Environmental intervention, Sodium, Salt, Potassium, Health literacy, Food literacy, Blood pressure, 24-h urine

## Abstract

**Background:**

Blood pressure is a primary cardiovascular disease risk factor. Population-wide governmental strategies aim to reduce lifestyle and dietary risk factors for hypertension, one of which is an unbalanced diet with high sodium and low potassium intakes. Nutrition interventions in the workplace are considered a promising approach in encouraging health-promoting behaviors. We developed and conducted the health promoting sodium reduction trial “Healthful & Tasty: Sure!” in worksites in the German-speaking part of Switzerland from May 2015 to Nov 2016, for which we present the study protocol and baseline characteristics.

**Methods:**

Healthful & Tasty, a cluster nonrandomized single-arm trial with calibration arm, aimed to demonstrate the effectiveness of a combined educational and environmental intervention in the workplace in reducing employees’ average daily sodium/salt intake by 15%. To this end, health and food literacy of employees and guideline compliance among the catering facility team needed to be improved. The primary outcome measure was sodium/salt intake estimated from sodium excretion in a 24-h urine sample. Secondary outcome measures included changes in the overall qualitative diet composition, blood pressure, anthropometric indices, and health and food literacy.

Of eight organizations with catering facilities, seven organizations took part in the nutrition education and catering salt reduction interventions, and one organization participated as a control. Overall, 145 consenting employees were included in the staggered, one-year four-phase trial, of which 132 participated in the intervention group. In addition to catering surveys and food sampling, the trial included five follow-up health assessments including questionnaires, blood pressure measurements, anthropometrics, and sodium, potassium, and iodine intake measurements obtained from 24-h and spot urine samples, and a food record checklist. Exploratory and hypothesis generating baseline statistical analysis included 141 participants with adequate 24-h urine samples.

**Discussion:**

Despite practice-driven limitations to the study design and small cluster and participant numbers, this trial has methodological strength and will provide important insights into the effectiveness of a combined educational and environmental intervention to reduce salt intake among female and male Swiss employees.

**Trial registration:**

German Clinical Trials Register, DRKS00006790. Registered 23 September 2014.

## Background

Stroke and ischaemic heart disease are the most common noncommunicable diseases and causes of disability and death worldwide [1, 2]. These cardiovascular diseases (CVD) impose a large economic burden: in Switzerland the annual direct health care costs attributable to CVD were estimated to be CHF 10.3 billion in 2011 [3].

About half of CVD events are estimated to be attributable to high blood pressure [4]. Yet many people are unaware of being hypertensive [5, 6], and strokes and ischaemic events often occur without prior diagnosis of hypertension [7]. Population-wide governmental strategies aim to lower dietary and other lifestyle risk factors for hypertension/CVD and promote protective factors [8, 9]. Experts acknowledge the health protective, blood pressure reducing role of a restricted sodium (Na) intake below an average of 2 g (87 mmol) per day [10, 11]. In addition, a sufficient potassium (K) intake (> 3.5 g (90 mmol)/day) and a dietary Na/K ratio below 0.6 by weight (< 1 by mmol) is a related public health goal [12]. Countries worldwide have established national programs for stepwise shifting of the overall population Na intake distribution [13] towards 30% lower intakes [14, 15].

Based on research on population Na intake [16, 17] and Na content of foods/catering offerings [18–20], the Swiss salt strategy defined an intermediate goal for Na intake reduction of at least 16% for the population in four years. That is, from an average of 3.5 g to below 3 g Na, or from 9 g to less than 8 g salt equivalent (short salt, NaCl) intake per day [21] -the final long-term target being 5 g/day [10]. The food industry, excepting food service (catering), became involved in a voluntary reformulation program [22] to reach defined Na goals (e.g., [23]). However, actions targeting eating behavior to reduce Na intake have not been pursued in the absence of consistent evidence of their effectiveness [24]. The national research program NRP69 Healthy Nutrition and Sustainable Food Production, which was seeking practical answers to how people in Switzerland can achieve a healthy diet, offered a framework to investigate feasible ways of Na intake reduction [25].

About three-quarters of the adult Swiss population eats lunch away from home [26], and almost 15% of the general population is estimated to frequent communal catering [27]. Persons regularly eating in staff canteens tend to follow a Western, unhealthy, diet [28], and structural catering initiatives were rated helpful in promoting healthy food choice [29]. The World Health Organization (WHO)/World Economic Forum [30] and the FAO Nutrition Division [31] encourage nutrition interventions in the workplace setting because women and men in the age range of 15 to 65 years can be reached repeatedly, and health-promoting behavior may thus be sustainably influenced. Moreover, Shain & Kramer [32] emphasized that only health promotion interventions in the workplace that address individual as well as environmental influences will be effective, and Eickolt et al. [33] argued that an environment -such as an organization with a staff canteen- conducive to learning supports enhancement of health literacy.

We thus developed a combined educational and environmental intervention in the workplace that aimed at reducing employees’ Na intake by 15% in one year and supposed that with enhancing generic and nutrition-specific health literacy participants’ social networks may also benefit from transfer of knowledge and skills.

## Methods

We followed the CONSORT guidelines for reporting trials [34].

### Study setting

The worksite-based intervention trial Gesund & Gut: Na Klar! (Healthful & Tasty: Sure!) was conceived for implementation in the German-speaking part of Switzerland. It was restricted to eight of the 26 Swiss cantons for language, logistical, and economic reasons. The experimental population consisted of female and male employees, aged 15 to 65 years, with access to small (≤ 150 luncheons/day), medium (151–500 luncheons/day), and large (> 500 luncheons/day) staff canteens of both public and private organizations.

### Study design

Healthful & Tasty is a combined educational and environmental trial in the Swiss workplace to promote health by lowering Na intake in a nutritionally balanced, healthy diet. Health promotion activities in the workplace are typically driven by legal responsibilities for occupational health and safety in Switzerland. Nutrition education and structural measures in catering facilities are not standard components in the national program Friendly Work Space [35]. Moreover, many organizations with staff canteens outsource catering and related food health responsibility to large catering firms, which offer labeled, nutritious menus. The trial recruited organizations and their employees, but catering firms had to agree to the intervention as well. We established a cluster randomized controlled trial (cRCT), received ethical approval by Swissethics, and registered the trial in September 2014.

Considering experiences from our own studies in communal catering [18, 19, 36, 37] and reports in the literature [38–41], and in agreement with the NRP69 scientific steering committee we prepared for having to adjust the study design if recruitment with randomization and matching at cluster level proved to be infeasible within six months (Table 1).

**Table 1:** Study design options of the “Healthful & Tasty: Sure!” workplace trial

	Experimental study design	Alternative quasi-experimental study design
Design	Cluster randomized controlled trial	Cluster nonrandomized single arm trial with calibration arm
Blinding	No	No
Pair-wise matching	Yes	No
Sample size estimation (power calculation)	Assumptions: baseline mean 24-h urine sodium excretion of 180 mmol [16, 17, 42], standard deviation of 65, intra-cluster correlation coefficient of 0.05, 16 clusters and drop-out rate of 25%. Objective: detect between-group difference of at least 15% (i.e. 27 mmol) at trial end with alpha 0.05 and 80% power.	Assumptions: baseline mean 24-h urine sodium excretion of 180 mmol [16, 17, 42], standard deviation of 65, intra-cluster correlation coefficient of 0.05, and drop-out rate of 10%. Objective: detect sodium intake reduction by 15% (i.e. 27 mmol) until trial end with alpha 0.05 and 80% power.
Numbers required to measure effectiveness	Number of clusters (participants, before 25% drop-out) 8 intervention clusters (*N* = 395, 50 per cluster) 8 control clusters (N = 395, 50 per cluster)	Number of clusters (participants, before 10% drop-out) 7 intervention clusters (*N* = 112, 16+ per cluster)

### Recruitment

Based on our power calculation for cRCT sample size, we estimated the need for eight intervention and eight control clusters, each with 50 participants per cluster to detect a between-group difference of at least 15% in mean 24-h Na excretion in urine at trial end with alpha 0.05 and 80% power [16, 17, 42] (Table 1). The two-stage cluster sampling started in November 2014. Following an intermediate recruitment evaluation in April 2015, we switched design as shown in Table 1. By then 60% of 389 potential clusters had been resolved, and seven organizations and their catering facilities had agreed to participate but did not accept random allocation to the intervention or control arm. The design change had no impact on sampling procedures and documents at the individual participant level, the ethics committee approved the change, and the clinical trial registration was updated and annotated. Recruitment at cluster level was stopped due to time constraints at the end of October, 2015, in virtue of having achieved the estimated minimum sample size (seven intervention clusters, at least 112 participants).

### Eligibility criteria, registration and consent

#### Cluster level

Participation was solicited from organizations via invitation letters to their respective managers that enclosed study information and a reply form with an individualized link for online reply. Inclusion criteria at cluster level were (1) organization with an on-site, meal producing staff canteen; (2) location in the canton of Bern, Solothurn, Aargau, Basel-Stadt, Basel-Land, Zurich, Zug, or Luzern; (2) at least 50 employees (exclusive of catering staff); and (3) the canteen sells at least 50 luncheons per day. All queried organizations that met these requirements, or that did not, as well as those refusing participation were asked if health promotion is part of the corporate mission in order to evaluate whether this influenced willingness or interest in participation. Indication of reason for refusal was optional. Interested organizations indicated general characteristics including kind of catering operation, number of employees, whether it was a shift operation, and whether it would accept random allocation to the intervention or control arm. After an on-site meeting with the investigator, organization and catering operation managers communicated their final decision for participation in the intervention or control arm. A gatekeeper agreement governed the rights, commitments, and cooperation between the organization, if applicable the catering firm, and the university. A study coordinator was designated at the organizational level, usually the person responsible for workplace health promotion.

#### Participant level

Operation management informed employees about study participation via email, intranet, and/or bulletin board and invited them to an on-site information session with the research group. A brief outline of the study included in the message listed the inclusion criteria: (1) men, and women who are not pregnant; (2) employment at the organization for at least one year from the beginning of the intervention in any job in the organization except a position in the staff canteen; (3) age 15–65 years; (4) eating on average twice a week or more in the staff canteen; (5) proficiency in German; (6) no kidney or cardiovascular disease, or medication intake or any other issue affecting urine collection or analysis; (7) no severe food intolerance or allergy that prevents food intake in the staff canteen. Attendees of the intervention sessions were given study information that included the consent form in duplicate, and a reply form with secure link for online registration. The same documents were disseminated to employees who did not attend. Within two weeks, employees registered using the reply form giving their name, age, professional position, contact information, etc., and also giving written, signed consent (if younger than 18 years, also by legal representative).

As permitted by the organizations, we conducted an additional, anonymous online nonresponder survey. It was combined with the study health questionnaire (see below) and assessed reasons for nonparticipation [43]. Results will be reported elsewhere.

### Study objectives

The primary objective was to demonstrate the effectiveness of a combined educational and environmental intervention in the workplace and staff canteen in reducing consumers’ mean daily total dietary NaCl intake by at least 15% in comparison to consumers served regular luncheons and having access only to general, publicly available information on healthy eating. Following the key health promotion approach of empowerment [44, 45] at organisational and individual levels, the secondary objectives were to improve the generic and nutrition-specific health literacy (HL) [46, 47] of employees and enhance guideline compliance among the catering facility team.

The study further pursued an exploratory objective: to validate both a Na- and K-specific food record checklist and a late afternoon spot-urine measurement against 24-h urinary excretion.

### Primary outcome measure

Changes in Na/NaCl intake (estimated from 24-h urinary Na excretion) in the intervention as compared with the control group.

### Secondary outcome measures

Changes in the overall qualitative diet composition (Na/K ratio), blood pressure, anthropometric indices, generic HL, and nutrition-specific HL in the intervention as compared with the control group. In addition, iodine (I) intake was monitored because table salt is fortified with iodine to ensure sufficient population intake in Switzerland [48].

Changes in Na/NaCl and K levels in luncheons offered daily and consumed regularly in the intervention catering facilities as compared with their levels in the control organisations’ catering facilities.

### Intervention design

**Fig. 1:**
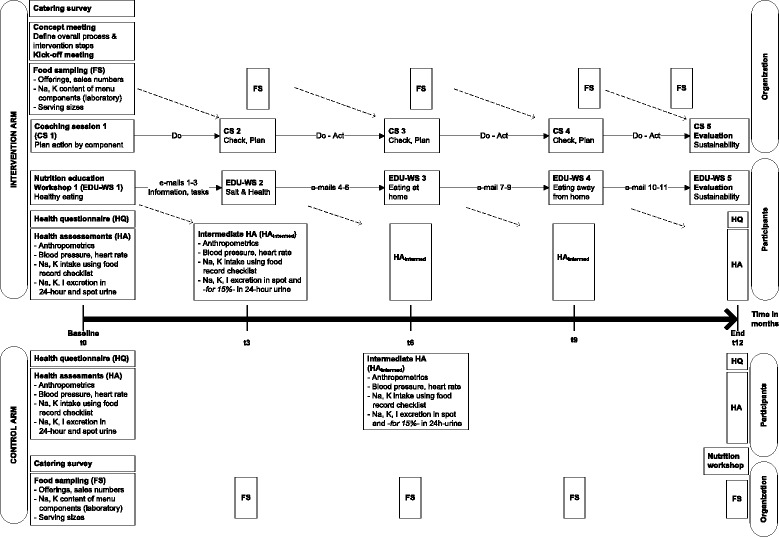
Flow diagram of intervention trial elements, baseline to study end

The staggered, four-phase (4 × 3 months) community trial over the course of one year in organizations with staff canteens combined educational and environmental interventions with regular follow-up measures in the intervention arm, compared to measures in organizations without intervention (control arm). Both the educational and environmental intervention programs covered five themes, implemented in four to five events, and included evaluation steps. They were matched with a maximum five follow-up measurements per person and canteen, at start/baseline (t0), after three (t3), six (t6), nine (t9), and 12 months (t12, end) (Fig. 1).

### Environmental intervention, theoretical background and program

With a view to develop and strengthen individual and environmental resources, the catering intervention aimed at creating a supportive nutrition environment for health in the workplace [49–51]. The coaching of catering teams was based on the awareness-to-adherence model [52] applied in the context of health risk management and it should enhance guideline compliance [53, 54]. The Hazard Analysis and Critical Control Points (HACCP) concept served as a model to control critical points of salt entry in luncheon production [18]. We hypothesized that becoming more knowledgeable and aware about food health risks and means of their practical management will empower catering staff to adopt the Swiss quality standards for health-promoting communal catering (SQSCC) [55]. We further expected it will motivate individuals and teams to commit to and routinely implement the standards for composition and production of a balanced, tasty food supply with controlled salt content (2.5 g salt per luncheon serving [55]).

The coaching program was structured in five sessions, repeatedly following the Plan-Do-Check-Act cycle for a process of continual quality improvement (Fig. 1). Aside from short presentations to impart knowledge and report results of laboratory food analysis, moderated workshops were conducted to analyze the initial position, plan in a participatory process small, feasible, and potentially the most effective changes to salt-critical menu components, and evaluate their success or failure after implementation. In addition, catering team members completed short questionnaires to follow up on their awareness, attitudes, and motivation at different times, and to evaluate the overall program and the coaching (see below, measurements). In the final workshop, coach and catering team discussed benefits and gains in knowledge and skills, program success, and the prospect of sustainability within the particular organizational conditions. A formally trained member of the research group who is an experienced chef and registered dietician implemented the coaching program [56].

### Educational intervention, theoretical background and program

We based the educational intervention on the concept of nutrition-specific HL, referred to as food literacy (FL), as suggested and described by Krause et al. [57]. The three functional, interactive, and critical forms of HL/FL [57–59] were to play a key role in the various stages of the dynamic Health Action Process Approach (HAPA) [60], which provided the underlying model to explain behavior change. We hypothesized that becoming more food literate will empower and motivate participants to change their individual eating behavior and actively involve their community of work colleagues, family, and friends when eating more healthily, thereby lowering their Na intake at the workplace and at home.

The education program was structured in five themed workshops, which were combined with intermediate email messages providing additional information, links to online nutrition games or films, and practical assignments (Fig. 1). Thus, continuous contact with participants was possible over the one-year intervention period, as recommended by Schwarzer [61]. Multicomponent interventions combining web-based components with in-person contact also have been shown to be effective [62, 63]. The program imparted knowledge on balanced diet and health, with a specific focus on salt as a dietary risk factor, and fostered practical abilities and skills related to food choice in and out of the home. Prior to the final workshop on program sustainability, participants completed an online, program-specific evaluation questionnaire (Fig. 1). Two experienced nutritionists who are registered dieticians and lecturers in dietetics realized the education program [64].

A description about development and implementation of the environmental and educational intervention will be published elsewhere.

### Data collection at cluster level

#### Catering survey

After registration, catering production managers completed a 57-item questionnaire [36] that served to characterize catering facilities by operational factors, offerings, and health promotion interests and activities. They also completed a quality evaluation checklist, rating the actual catering offerings in comparison with the SQSCC [36, 65]. The information helped the coach to understand specific organizational conditions and prepare the intervention activities.

Three short questionnaires were established and applied to follow up on the guideline compliance dimensions that were targeted by the environmental program (Fig. 1) [66]: (1) the awareness of catering staff of the SQSCC and salt-health issues at t0; (2) their intermediate attitude and motivation towards implementing planed changes and continue intervention measures in agreement with the standards (t3), and (3) to evaluate the environmental program and coaching overall at t12.

#### Intervention actions and food sampling

The intervention focused on lunch as the main meal most commonly consumed in business staff restaurants, and the most frequently purchased and consumed offerings. These were different types of plated, multicomponent menus such as meat or fish, daily special, vegetarian, etc., which could be supplemented by a soup or menu salad [18]. We chose a stepwise improvement process, planning targeted changes in food production per menu component, which typically were (1) a nondairy meat, fish, or plant protein component; (2) a carbohydrate, starchy foods component; and (3) a vegetable or menu salad component. These components were either presented separately on a plate or as part of a composite dish (e.g., lasagna). Supplementary components on a different food base included (4) gravy/sauces and (5) dairy products like grated cheese or any other kind of topping. The different plated menus as well as self-service, buffet-style offerings would thus be broadly affected by Na/NaCl reduction measures. Actions taken to reduce Na/NaCl could involve the entire operating sequence that is menu-planning, purchase/use of specific products, recipes, production, serving procedures, and more.

Food sampling at five times (t0 to t12) included all individually produced components of plated menus offered on sampling dates. Depending on the implemented intervention actions, additional food samples may have been taken. Single food items were sampled as end products prior to serving, in specifically labeled containers to allow identification of organization, follow-up date, type or name of food, and menu source. Standard serving size was determined by weighing servings set by restaurant managers and served by staff. For all buffet-style and additional food items without fixed serving sizes, reference values were used from the food record checklist (see below, data collection at participant level). Sales numbers of the food items were recorded on each day food was sampled.

#### Laboratory analysis of sodium and potassium concentrations in food

Study staff delivered catering food samples in labeled containers on the day of production to Federal Food Safety and Veterinary Office (FSVO) laboratories. Upon delivery, the samples were homogenized using a laboratory mixer and, depending on day of analysis, were kept cooled (4 °C) or deep-frozen (− 20 °C). The study lab technician weighed duplicate portions of the food samples for Na and K extraction with 1% nitic acid (at 90 °C) and subsequent analysis; approximately 40 g were retained deep-frozen for repeat or additional measurements. Na and K (mg/kg of fresh mass) were determined through inductively coupled plasma optical emission spectrometry (ICP-OES; 589.5 nm Na; 766.4 nm K; iCAP 7400 dual view, Thermo). The accuracy and precision of the ICP-OES determinations were checked periodically with National Institute of Standards and Technology (Gaithersburg, MD, USA) standard reference material SRM1548a-Typical Diet. The measurements of Na and K values in the reference material agreed well with the certified values. For additional, independent quality control and evaluation purposes, FSVO laboratories used an argentometric method to determine chloride (Cl) in 24 selected menu components that were uniformly distributed over the concentration range. Linear regression analysis of Cl vs. Na (R^2^ = 0.98) showed that the 95% confidence interval of the slope included the theoretical Cl/Na atomic weight ratio of 1.54, which indicated that Na and Cl content were correlated and Na therefore well represents NaCl content.

#### Estimation of sodium and potassium content of menu components and servings

Na and K concentrations of menu components in mg/kg were converted to g per 100 g (%) and to g per serving. The equivalent NaCl content was calculated by multiplying Na content by 2.54, the molar weight ratio of NaCl (58.5 mg/mmol) to Na (23 mg/mmol). Data for served menu components were added to represent the overall Na/NaCl and K contents of specific served, plated menus. Serving sizes, Na, NaCl, and K contents were weighted by menu sales numbers to understand the respective impact upon employees’ intakes.

### Data collection at participant level

#### Health questionnaire

Participants as well as nonresponders filled out a health questionnaire comprised of 68 questions at t0, and participants did so again at t12. The questionnaire assisted characterization of study participants and nonresponders, and captured changes in secondary outcome measures and specific items related to the education program. It covered seven areas: demographic and socioeconomic position, health status, health in everyday life, and the health behaviors eating and drinking, physical activity, and smoking. It was largely based on existing, validated questionnaires to allow for data comparability with other international surveys [66]. Core questions inquired about generic HL and nutrition-specific HL/FL, salt awareness, and nutrition self-efficacy. Given the workplace health promotion setting of our trial, we restricted assessment of generic HL to the set of items under the health promotion domain of the validated European Health Literacy questionnaire (HLS-EU-Q47, in German) [67, 68], and calculated the HL index (0 to 50 points) according to the HLS-EU Consortium recommendation [69]. HL was categorized as inadequate (25 or less), problematic (> 25–33), sufficient (> 33–42), and excellent (> 42–50). For measurement of nutrition-specific HL/FL, we integrated a set of 16 items in the questionnaire, which underwent two-stage testing [70]. Validation [71] revealed 12 of the items suitable to reliably represent basic elements of FL and form a practical short questionnaire (SFLQ) for which a FL score (7–52, lower score for lower FL) was established.

The questionnaire could be completed in paper form or filled in online. Study staff checked the returned/submitted questionnaires for completeness and inconsistencies, and followed up with participants to obtain missing answers.

#### Health assessments

As shown in Fig. 1, participants from organizations in both study arms were followed-up at their workplaces every three months, from t0 to t12. In a short follow-up of 20 min per participant, three experienced and trained dieticians/nutritionists instructed participants about urine collection, handed out study materials, and used portable calibrated instruments to obtain body weight, height, waist and hip circumference, blood pressure, and heart rate measurements. As of t3, and in accordance with ethical guidelines, participants were asked to report any adverse events relating to study participation. Measurements were recorded on result sheets that were given to participants for personal information after data entry into the trial database.

#### Sodium, potassium, and iodine intake measurements

We repeated a three-day measurement protocol to estimate Na, K, and I intakes during the one-year intervention period. The protocol combined 24-h and spot urinary excretion with questionnaire measures as suggested elsewhere [72, 73]. Because 24 h of urine mostly reflects nutrient intake one to two days prior to collection, we applied a Na- and K-specific food record checklist (FRCL) on days one to three to complement collection of a spot-urine on day two, and a 24-h urine collection on day three. We were aware of the burden of repeated 24-h urine collections, and the related risk of dropouts, and therefore we requested all participants to collect urine for 24 h at t0 and t12, but only a 15% random sample to collect 24-h urine at t3, t6, and t9. Everyone had to complete the FRCL and provide spot urine collections, though.

#### Food record checklist

A semi-quantitative Na- and K-specific FRCL was filled out on three previously agreed on, consecutive days to complement collection of spot- and 24-h urine with qualitative information on Na and K food sources and their daily consumption frequency, discretionary salt use at time of eating, and food/menu choices and sensory rating in the staff canteen. This dietary assessment instrument [74] was newly developed and underwent iterative two-stage testing prior to use. Details will be published elsewhere, in the context of the validation study (see above, explorative objective). Briefly, the FRCL is a grid constructed by a closed-ended list of 13 food categories and relevant subgroups with related, typical add-ons, which represent the major sources of Na and K. Frequency and quantity of daily intake is reported by ticking the number of reference portions (1 to 5+) consumed per meal (breakfast, AM snack, lunch, etc.). For estimation of Na and K intake, a FRCL database was compiled based on the Swiss food composition database [75] and FSVO laboratory data from earlier studies [18, 19]. Additional questions on meal-based discretionary salt use and aspects of eating in the staff canteen were added at the end of the checklist.

Study staff instructed participants about how to complete the FRCL. At time of pick-up, together with urine containers, study staff collected the three checklists and checked them for completeness and inconsistencies. If needed, participants were contacted to verify information prior to data entry in the trial database.

#### 24-h urine collection

Participants collected urine following the WHO/PAHO standard protocol during a previously agreed upon 24 h period [72]. Study staff instructed participants individually on the urine collection procedure. They received a bag carrying two labeled collection bottles (2 L, 3 L) and a urine collection recording sheet. On the day of collection, the first morning urine was discarded (start time) and all urine voided thereafter was collected up until next morning at approximately 24 h after start (finish time). Participants recorded collection start and finish dates and times, missed/spoiled urine collections, and medication use. They were asked to keep the bottles dark and cool until pick-up and transport to the centrally based laboratory.

#### Spot urine collection

Participants collected a timed, midstream urine void near the midpoint of a morning-to-morning 24-h collection period, at around 17 h00 [76]. Study staff instructed each participant on collection procedure and completion of the recording sheet, which asked for the date and actual time of spot-urine collection, the time and kind of last solid and liquid food intake, the type and intensity of physical activities during the four hours prior to voiding, and medication use. These information will be considered when comparing spot- and 24-h urine based Na/NaCl intakes. Participants used a labeled 100 ml screw capped container for spot-urine collection, which they kept refrigerated and dark in a 1 L plastic zip-top bag until pick-up.

Study staff collected all urine containers and recording sheets, checked the latter for completeness, and if needed contacted participants to verify information prior to data entry in the trial database.

#### Laboratory determination of urinary sodium, potassium, creatinine, and iodine concentrations

The Dr. Risch medical laboratory collaborated with the FSVO laboratories in central urine analysis in Bern, Switzerland. Study staff delivered 24-h and spot urine containers to the medical laboratory. A technician gravimetrically estimated the individual total 24-h urine collection volume in milliliters assuming a specific weight of one. For both 24-h and spot urine samples five 2 ml aliquots each were prepared. Two aliquots of each sample were analyzed immediately; the remaining aliquots were retained at − 20 °C for later analysis.

The medical laboratory analyzed urinary Na and K concentrations (mmol/L) with an indirect ion selective electrode technique (Roche eLabDoc ISE indirect Na-K-Cl for Gen.2 Global, V9.0). Urinary creatinine (Creat) concentration (μmol/L) was determined by the kinetic Jaffe method (Roche eLabDoc Creatinin Jaffé Gen.2, V17.1). The Dr. Risch medical laboratory performed daily internal and external quality controls according to SWISSMEDIC and QUALAB guidelines.

If urinary Na concentration was below detection limit (20 mmol/L), the FSVO laboratories analyzed a retained aliquot using ICP-OES, which is more sensitive than ISE. They further determined urinary I concentration (μg/L) with inductively coupled plasma mass spectrometry using isotope dilution analysis with addition of an I-129 tracer solution and measuring the I-127/I-129 ratio [77] to calculate I concentration according to Adriaens et al. [78]. In each series of Na and I measurements, four samples of the standard reference material 2670a (US National Institute of Standards and Technology) were measured for quality control purposes. All measured values agreed well with the certified Na and I concentrations.

#### Estimation of daily sodium, potassium, and iodine excretion/intake

We standardized urinary measures to a 24-h period by correcting the urinary volume for the reported duration of urine collection. Daily Na and K excretion (mg/24 h) was derived by multiplying urinary concentration (mmol/L) by 24-h volume (L/24 h) and by atomic weights (Na, 23 mg/mmol; K 39.1, mg/mmol). Predicting Na and K intake from urinary excretion without correcting for possible minor losses via sweat or feces has been judged feasible and to have good predictive power [79]. For equivalent daily NaCl intake (g/day), we multiplied Na excretion (mg/24 h) by 2.54. Calculation of daily I intake (μg/24 h or μg/day) accounted for urinary concentration that represents 92% of ingested iodine [80, 81]: urinary concentration (μg/L) / 0.92 × 24 h-volume (L/24 h).

#### Completeness of 24-h urine samples

We accepted collection durations of 24 h +/− 4 h (20-28 h), but rejected urine samples if (i) total volume was below 300 ml, (ii) two or more voidings were missed, and (iii) urinary Creat excretion was above 400 μmol/kg/24 h. After exclusion of unacceptable urine samples, we compared the measured 24-h Creat excretion (μmol/kg/24 h) of remaining samples with sex-specific Swiss reference values [82] to evaluate their quality (adequate, incomplete, or over-collected). We checked plausibility of Creat concentrations of incomplete and over-collected samples, using explanatory information assessed at time of urine collection, about body mass index (BMI) and waist circumference (adiposity/low lean body mass), metabolic disease, intensity of physical activity, and food/protein intake. In a subsequent sensitivity analysis, we compared mean Na and K excretion and NaCl intake of the inadequate and adequate samples, the inadequate and all acceptable samples, and all acceptable samples with those of the adequate samples. We tolerated relative differences of maximum +/− 15% to include all acceptable samples in analysis [83].

#### Blood pressure

Blood pressure (BP) was measured according to the 2013 European guidelines for office BP management [84]. We used new, calibrated OMRON HEM-907 validated automatic BP measuring devices. The device additionally performed heart rate measurement. Participants were seated in the quiet room and at rest for ca. 5 min prior to measurement. Depending on upper arm circumference, a different cuff size (S/M/L) was chosen. At t0 we first performed single BP readings on both arms to determine the arm for all follow-up measurements. If a between-arms difference in systolic BP of > 10 mmHG was identified, the arm with the higher value was taken as reference arm, otherwise we performed measurements on the non-dominant arm. We took three consecutive measurements one minute apart and calculated the mean of the last two readings. Hypertension was defined as systolic BP ≥140 mmHg and/or diastolic BP ≥90 mmHg and/or being on antihypertensive treatment.

#### Anthropometrics

Anthropometric measurements followed a protocol that was adapted from international standard procedures and applied in other surveys in Switzerland [85, 86]. Study staff briefly explained all measures to the participant and asked for approval. Participants wore light clothing and no shoes when body weight and height were measured to the nearest 0.1 kg and 0.1 cm using a digital flat scale (seca 803) and a mobile stadiometer (seca 217). Before entering data into the study database, we subtracted 1.0 kg from the body weight readings in order to account for average weight of garments [85, 87]. Waist circumference was measured with the shirt lifted, halfway between the iliac crest and the lower rib, using an ergonomic circumference measuring tape (seca 201). The measure was taken twice to the nearest 0.1 cm. At a difference of more than 0.5 cm, a third measurement was taken, and the mean of the two closest measurements calculated. In analysis we considered two measures of weight status and related cardio-metabolic risk, BMI, calculated as weight divided by height squared (kg/m2) [88] and the waist to height ratio (WHtR) [89].

### Data management

Study data collection and management was arranged in REDCap (Research Electronic Data Capture), a secure, web-based application designed to support data capture for research studies, hosted at the Clinical Trials Unit (CTU), University of Bern [90].

For urinary and food laboratory analysis data, partners defined a common database structure and transfer protocol between the laboratories’ information and management systems and the trial’s database management system. At time of data import into REDCap, we checked for keyboarding errors, completeness and consistency of data, and made corrections in all systems, if applicable. CTU and study staff verified all imports in REDCap.

A trained research assistant entered the health questionnaire (paper version), FRCL, urinary collection recording sheets, and catering surveys in REDCap. Data entry was double checked by study staff.

### Statistical analysis

For baseline analysis, continuous variables were summarized descriptively using mean and 95% confidence interval (CI) or median and range depending on sample size and type of variable. For categorical variables, we used frequency and percentage. Variables were summarized overall, separately for intervention and control group, and by sex. At cluster level, Na, K, and NaCl levels of menu components were calculated weighted for sales numbers. At participant level, comparisons between groups were performed using t-tests for continuous variables, and chi-squared tests or Fisher’s exact tests for categorical variables depending on the expected number per cell. We applied two-tailed tests with significance level 0.05 for all analyses. Because baseline analysis was exploratory and hypothesis generating, no adjustment for multiple testing was done.

We used and will use baseline data to explore factors related to salt intake and evaluation of questionnaires. Statistical analyses are described in the respective articles [71, 91]. Future analysis will include application of a linear mixed model with organization as random effect to assess the change in primary outcome from t0 to t12. All analyses are run using R 3.3.2 [92].

### Dissemination

We will make findings known within the scientific community and public health practice. After intervention closure (November 2016), an exchange of experiences for identifying barriers and promotors took place, which involved participating organizations and stakeholders. This workshop was intended to inform guarantors of the Swiss nutrition strategy about a possible implementation model in the workplace setting. FSVO will make the coaching and nutrition education manuals and related tools [56, 64] accessible online to their implementation partners and other interested health professionals.

### Baseline information

Presentation of baseline data is restricted to enrollment and follow-up numbers, and basic characteristics of organizations and participants. All results of baseline analysis are published elsewhere.

### Participation

**Fig. 2:**
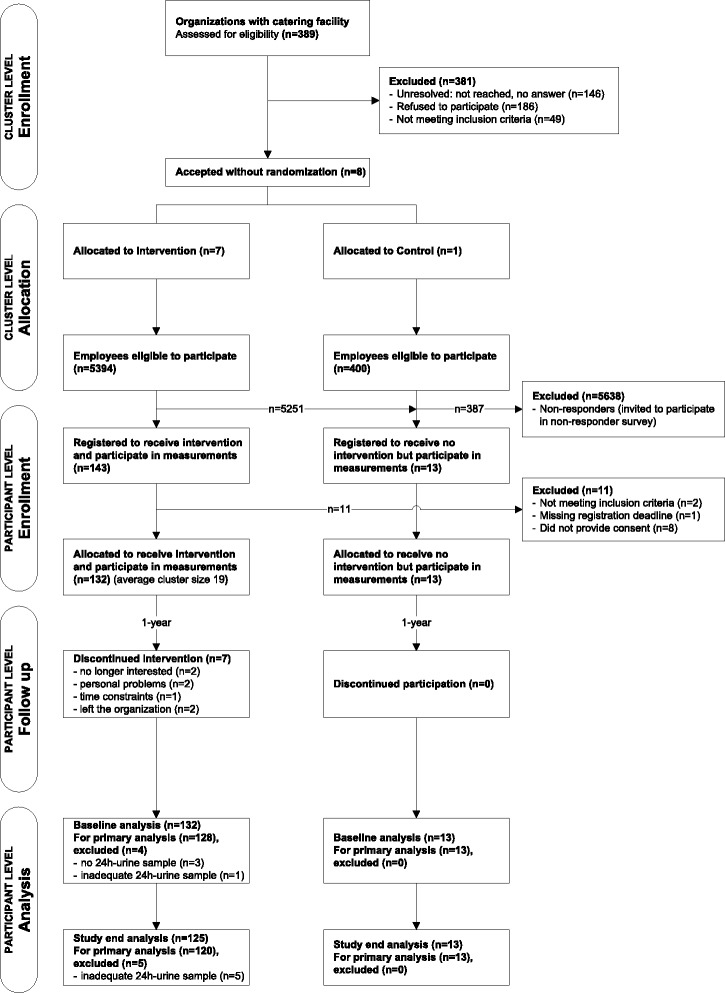
Trial flow diagram from enrollment to analysis at cluster and participant level

Figure 2 diagrams process of recruitment and enrollment through analysis at cluster and participant level.

#### Cluster level

A total of 389 organizations with catering facilities were assessed for eligibility. Of these, 146 were not reached, 186 declined to participate, and 49 did not meet the inclusion criteria. Finally, eight organizations were included in the trial, seven participating in the intervention arm and one in the control arm.

#### Participant level

Of 5794 eligible employees (5394 intervention, 400 control), 145 employees (132 intervention, 13 control) met the inclusion criteria and provided written consent. Baseline analysis included 141 participants (128 intervention, 13 control) with adequate 24-h urine samples.

### Baseline characteristics

#### Cluster level

Table 2 shows characteristics of participating organizations and their catering facilities, overall and by study arm. The public and private organizations were of medium to large size, with slightly more male employees, and the majority of organizations said they supported health promotion activities.

**Table 2:** Baseline characteristics of organizations and their catering facilities, overall and by study arm

		All	Intervention group	Control group
Number organizations	N	8	7	1
Manufacturing and service industry	n	2	2	–
Administration and offices	n	2	1	1
University and research institutions	n	2	2	–
Social service and welfare institutions	n	2	2	–
Health promotion is part of corporate philosophy	n	6	6	0
Total number employees eligible to participate	N	5794	5394	400
Median number employees eligible to participate	Median (range)	684 (228–1380)	868 (228–1380)	400^a^
% Women	Median (range)	48.5% (19% – 70%)	56% (19% - 70%)	37%
% Men	Median (range)	51.5% (30% – 81%)	44% (30% - 81%)	63%
Kind of catering facility				
Corporate entity	n	2	2	0
Outsourced	n	6	5	1
Number luncheons sold per day overall (incl. buffet)	Median (range)	214.5 (128–636)	232 (128–636)	161
% Plated menus (all kind)	Median (range)	83.9% (76.1% - 91.8%)	85% (76.1% - 93.9%)	76.4%
% Self-service buffet	Median (range)	16.1% (8.2% - 23.9%)	15% (6.1% - 23.9%)	23.6%
Size of catering facility (sales numbers)				
Small (< 150 luncheons/day)	n	2	2	0
Medium (150–500 luncheons/day)	n	5	4	1
Large (> 500 luncheons/day)	n	1	1	0
Catering system (*multiple answers*)				
cook-serve	n	7	6	1
cook-hot hold	n	7	7	0
cook-chill	n	6	6	0
cook-freeze	n	1	1	0
Processing/convenience level of foods/products (*two answers/most frequent per organization*)				
level 0 (unprocessed foods)	n	4	4	0
level 1 (ready-for-kitchen)	n	5	4	1
level 2 (ready-to-cook)	n	4	4	0
level 3 (ready-to-end-cook)	n	1	0	1
level 4 (ready-to-heat)	n	2	2	0
level 5 (ready-to-eat)	n	0	0	0
Number catering staff	N	139	131	8
Qualified	n	84	79	5
Unqualified	n	55	52	3
Catering staff knows	N	60	54	6
Swiss quality standards for health-promoting communal catering	n	40	35	5
Recommended salt intake (< 5 g/day)	n	26	23	3

^a^Absolute number since N = 1

All of the organizations except two social service/welfare institutions outsourced catering. Kitchens were characterized by a mix of catering systems that implied use of foods of varying convenience levels. Across all catering staff, 60% had formal certification in food preparation. Two-thirds reported knowing the SQSCC [55], and over half did not know the WHO recommendation for salt intake [10]. The staff canteens offered between two and four different menus (including buffet style) for lunch; the standard plated menus were the most frequently consumed (83.9%).

#### Participant level

Table 3 provides data on demographic and socioeconomic characteristics of all participants and by study arm.

**Table 3:** Baseline demographic and socio-economic characteristics of participants, overall and by study arm

		All	Intervention group	Control group
Number participants	N	141	128	13
Women	n (%)	70 (49.6%)	65 (50.8%)	5 (38.5%)
Men	n (%)	71 (50.4%)	63 (49.2%)	8 (61.5%)
Age (years)	Median (range)	46 (21–61)	46 (21–61)	48 (30–59)
	Mean (95% CI)	44.6 (42.8, 46.4)	44.1 (42.2, 46)	49.3 (44.6, 54)
15–34	n (%)	32 (22.7%)	31 (24.2%)	1 (7.7%)
35–44	n (%)	24 (17.0%)	23 (18.0%)	1 (7.7%)
45–54	n (%)	53 (37.6%)	45 (35.2%)	8 (61.5%)
55–65	n (%)	32 (22.7%)	29 (22.7%)	3 (23.1%)
Nationality				
Swiss	n (%)	119 (84.4%)	107 (83.6%)	12 (92.3%)
Non-Swiss	n (%)	22 (15.6%)	21 (16.4%)	1 (7.7%)
Household size	Median (range)	3 (1–6)	3 (1–6)	3 (1–6)
	Mean (95% CI)	2.6 (2.4, 2.8)	2.5 (2.4, 2.7)	2.8 (2, 3.7)
Household type (or structure)				
Single	n (%)	23 (16.3%)	21 (16.4%)	2 (15.4%)
Single or couple *with* children	n (%)	65 (46.1%)	58 (45.3%)	7 (53.8%)
Two or more adults *no* children	n (%)	53 (37.6%)	49 (38.3%)	4 (30.8%)
Education				
Primary/obligatory	n (%)	2 (1.4%)	2 (1.6%)	0 (0.0%)
Secondary	n (%)	35 (24.8%)	34 (26.6%)	1 (7.7%)
Tertiary	n (%)	104 (73.8%)	92 (71.9%)	12 (92.3%)
Working position				
Trainee	n (%)	3 (2.1%)	3 (2.3%)	0 (0.0%)
White and blue collar workers	n (%)	61 (43.3%)	52 (40.6%)	9 (69.2%)
Lower and middle management	n (%)	67 (47.5%)	63 (49.2%)	4 (30.8%)
Top management	n (%)	10 (7.1%)	10 (7.8%)	0 (0.0%)
Worktime equivalent (% Full Time Equivalent FTE)	Median (range)	100 (40–100)	100 (40–100)	90 (60–100)
	Mean (95% CI)	90.4 (88.1, 92.7)	90.8 (88.4, 93.3)	86.5 (78.8, 94.3)
Eating lunch at staff restaurant (times/week)				
0–1 times/week	n (%)	30 (21.3%)	30 (23.4%)	0 (0.0%)
2–3 times/week	n (%)	70 (49.6%)	64 (50.0%)	6 (46.2%)
4–7 times/week	n (%)	41 (29.1%)	34 (26.6%)	7 (53.8%)

The proportions of men and women were similar, and the mean age was 44.6 years. Most participants were Swiss, and about half lived in households with children. Participants were well educated, and the majority held a management position. Over half of participants worked full time, and 80% indicated they ate lunch at least twice a week in the staff canteen.

## Discussion

This quasi-experimental trial will provide important insights into the effectiveness of a combined educational and environmental intervention to reduce Na/NaCl intake among Swiss employees by at least 15% (primary outcome). Additionally, we expect changes in the Na/K ratio, blood pressure, anthropometric indices, HL, FL, and the Na and K contents of canteen luncheons (secondary outcomes).

### Strength and limitations

A major strength of this study is that it follows a two-pillar strategy that combines intervention at individual behavioral and structural levels in a workplace setting based on the social ecological model for public health interventions [93]. Multicomponent interventions that target changing behavior and practice of professionals were found to be effective and assist the creation of health-promoting environments [94]. Combined interventions are regarded as a promising approach to promote a healthy diet, improve nutrition knowledge [15, 30, 95, 96], and reduce salt intake [24].

Another important strength is the use of a complementary set of self-reported (subjective) and laboratory (objective) methods at cluster and participant level, covering a wide range of variables, which will allow meaningful interpretation of results [97]. Twenty-four hour urine collection is the standard approach to measurement of Na/NaCl, K, and I intake [72]. We recognize that any single urine collection may not be representative of usual individual intake. However, this method is considered an acceptable proxy for intake at the group level [98]. Obtaining complete 24-h urine collections every three months during a one-year intervention is rare and remains challenging [99]. Moreover, repeatedly combining different methods (24-h urine, spot urine, and FRCL) for the same person may help to identify alternative approaches to measuring Na and K intake with less burden and lower cost. To this end, a validation study is underway comparing Na and K measures from FRCL and spot urine with 24-h urine measurements.

Our experience showed that implementing intervention trials in the workplace is highly sensitive to how the real-world setting meets scientific demands [100, 101]. We chose a cRCT, which is commonly viewed as the most reliable design to assess the effectiveness of interventions and avoid bias [102]. However, the enrollment rate of organizations in our trial was much lower than expected, and interested organizations did not accept being randomized to intervention or control arms, which confirms observations made by Kwak et al. [39] who recognized randomization as one of the most important reasons why organizations refuse to participate in trials. For these reasons, and time constraints, we adjusted to a quasi-experimental study design. The final, cluster nonrandomized single-arm trial with calibration arm still allows following all participants and estimating intervention effectiveness with sufficient robustness [41, 103]. Participation at individual level was modest, but we anticipated a high follow-up rate since most participants seemed committed to participating in education workshops during the workday, and receiving regular measurement results of blood pressure, weight, Na and K intake, etc., as well as coupons for eating in the staff restaurant as compensation at study end.

We will use the RE-AIM (reach, effectiveness, adoption, implementation, and maintenance) framework [104] to estimate the trial’s final overall impact. Finally, we recognize that study findings can only be applied to similar organizations, not to the overall working population in Switzerland, since only certain German-speaking cantons were included and sample size was small.

## Abbreviations

BMI: Body mass index
BP: Blood Pressure
Cl: Chloride
cRCT: Cluster randomized controlled trial
Creat: Creatinine
CVD: Cardiovascular disease
FL: Food literacy
FRCL: Food record checklist
FSVO: Federal Food Safety and Veterinary Office
HL: Health literacy
HLS-EU: European health literacy survey
I: Iodine
ICP-OES: Inductively coupled plasma-optical emission spectrometry
ISE: Indirect ion selective electrode technique
K: Potassium
Na: Sodium
NaCl: Salt, salt equivalent
SQSCC: Swiss quality standards for health-promoting communal catering
t0: baseline
t12: study end
t3/t6/t9: follow-up at 3, 6, 9 months
WHO: World Health Organization
WHtR: Waist-to-height Ratio

## References

[1] World Health Organization (WHO). Global Health Estimates 2015: Deaths by Cause, Age, Sex, by Country and by Region, 2000–2015 2016 http://www.who.int/healthinfo/global_burden_disease/estimates/en/index1.html. Accessed 25.10.2017.

[2] World Health Organization (WHO). Global Health Estimates 2015: Disease burden by Cause, Age, Sex, by Country and by Region, 2000–2015 2016 http://www.who.int/healthinfo/global_burden_disease/estimates/en/index2.html. Accessed 25.10.2017.

[3] Wieser S, Tomonaga Y, Riguzzi M, Fischer B, Telser H, Pletscher M, et al. Die Kosten der nichtübertragbaren Krankheiten in der Schweiz. Schlussbereicht im Auftrag des Bundesamts für Gesundheit (BAG), Abteilung Nationale Präventionsprogramme. Bern: BAG; 2014.

[4] Lawes CM, Vander Hoorn S, Rodgers A (2008). International Society of Hypertension. Global burden of blood-pressure-related disease, 2001. Lancet.

[5] Guessous I, Bochud M, Theler JM, Gaspoz JM, Pechere-Bertschi A (2012). 1999-2009 trends in prevalence, unawareness, treatment and control of hypertension in Geneva, Switzerland. PLoS One.

[6] Murphy CM, Kearney PM, Shelley EB, Fahey T, Dooley C, Kenny RA (2016). Hypertension prevalence, awareness, treatment and control in the over 50s in Ireland: evidence from the Irish longitudinal study on ageing. J Public Health (Oxf).

[7] Word Health Organization (WHO) (2013). A global brief on hypertension. Silent killer, global public health crisis. World Haelth day.

[8] World Health Organization (WHO). Global status report on noncommunicable diseases 2014. Geneva: WHO; 2014. 10.1161/STROKEAHA.115.008097 25873596

[9] Bundesamt für Gesundheit (BAG) und Schweizerische Konferenz der kantonalen Gesundheitsdirektorinnen und -direktoren (GDK) (2016). Nationale Strategie Prävention nichtübertragbarer Krankheiten (NCD-Strategie) 2017–2024. Bern: BAG.

[10] World Health Organization (WHO) (2012). Guideline: sodium intake for adults and children. Geneva: WHO.

[11] Cogswell ME, Mugavero K, Bowman BA, Frieden TR (2016). Dietary sodium and cardiovascular disease risk - measurement matters. N Engl J Med.

[12] World Health Organization (WHO) (2012). Guideline: potassium intake for adults and children. Geneva: WHO.

[13] Rose G (2008). Rose's strategy of preventive medicine.

[14] World Health Assembly - World Health Organisation. Sixty-sixth world health assembly WHA66.10. Follow-up to the political declaration of the high-level meeting of the general assembly on the prevention and control of non-communicable diseases. Geneva: WHO. May 2013:27.

[15] World Health Organization (WHO). Global action plan for the prevention and control of noncommunicable diseases 2013-2020. Geneva: WHO; 2013.

[16] Chappuis A, Bochud M, Glatz N, Vuistiner P, Paccaud F, Burnier M (2011). Swiss survey on salt intake: main results. Lausanne: Lausanne University Hospital (CHUV).

[17] Beer-Borst S, Costanza MC, Pechère-Bertschi A, Morabia A (2009). Twelve-year trends and correlates of dietary salt intakes for the general adult population of Geneva, Switzerland. Eur J Clin Nutr.

[18] Beer-Borst S, Sadeghi L. Salz in der Gemeinschaftsgastronomie: Massnahmen zur Reduktion (salt in communal catering: reduction measures). Report on behalf of the federal office of public health. Bern: Berner Fachhochschule, Fachbereich Gesundheit, aF&E Ernährung und Diätetik; 2011.

[19] Sadeghi L, Beer-Borst S, Bürgisser P, für das Projektteam. Optimierung des Angebots der Gemeinschaftsgastronomie zur Reduktion des Salzkonsums in der Schweizer Bevölkerung. 2013. https://pdb.bfh.ch/search/pdbwebviewdetail.aspx?lang=de&projectid=aae1e33b-4a89-4b65-b985-72700845bac0&instId=3898707e-4722-4b93-9c06-c0a2784a7ab1. Accessed 25.10.2017.

[20] Zülli S, Allemann C (2011). Reduktion des Salzkonsums: Reduktion des Salzgehalts in verarbeiteten Lebensmitteln (reduction of salt consumption: reduction of the salt content in processed foods). Report on behalf of the federal office of public health.

[21] Federal Office of Public Health. Salt strategy for 2013 - 2016. Paper on a strategy for reducing salt consumption. Bern, Switzerland: Federal Food Safety and Veterinary Office; 2013.

[22] Bundesamt für Gesundheit (BAG). actionsanté - eine Initiative des BLV und des BAG. www.actionsante.ch. Accessed 25.10.2017.

[23] Bundesamt für Lebensmittelsicherheit und Veterinärwesen (BLV). Medienmitteilung vom 8.08.2015: Salzgehalt in Schweizer Broten konnte gesenkt werden. 2015. https://www.admin.ch/gov/de/start/dokumentation/medienmitteilungen.msg-id-58497.html. Accessed 25.10.2017.

[24] Trieu K, McMahon E, Santos JA, Bauman A, Jolly KA, Bolam B (2017). Review of behaviour change interventions to reduce population salt intake. Int J Behav Nutr Phys Act.

[25] Swiss National Science Foundation (SNSF). National Research Programme (NRP 69) Healthy Nutrition and Sustainable Food Production. 2013. http://www.nfp69.ch/en/projects/how-can-people-achieve-a-healthy-diet/project-salt-consumption. Accessed 25.10.2017.

[26] Bundesamt für Lebensmittelsicherheit und Veterinärwesen (BLV). Medienmitteilung vom 16.03.2017: Die Bevölkerung der Schweiz ist unausgewogen. 2017. https://www.blv.admin.ch/blv/de/home/dokumentation/nsb-news-list.msg-id-66016.html. Accessed 21.08.2017.

[27] Schweizerischer Verband für Spital-, Heim- und Gemeinschaftsgastronomie (SVG). Ein starker Verband für eine starke Branche. http://www.svg.ch/portraet. Accessed 03.03.2018.

[28] Kjollesdal MR, Holmboe-Ottesen G, Wandel M (2011). Frequent use of staff canteens is associated with unhealthy dietary habits and obesity in a Norwegian adult population. Public Health Nutr.

[29] Geaney F, Harrington J, Fitzgerald A, Perry I (2011). The impact of a workplace catering initiative on dietary intakes of salt and other nutrients: a pilot study. Public Health Nutr.

[30] World Health Organization, World Economic Forum. Preventing noncommunicable diseases in the workplace through diet and physical activity (2008). WHO/world economic forum report of a joint event. Geneva: WHO.

[31] Hawkes Corinna. Promoting healthy diets through nutrition education and changes in the food environment: an international review of actions and their effectiveness. Rome: Nutrition Education and Consumer Awareness Group, Food and Agriculture Organization of the United Nations 2013.

[32] Shain M, Kramer DM (2004). Health promotion in the workplace: framing the concept; reviewing the evidence. Occup Environ Med.

[33] Eickholt C, Hamacher W, Lenartz N (2015). Gesundheitskompetenz im Betrieb fördern - aber wie?. Bundesgesundheitsbl Gesundheitsforsch Gesundheitsschutz.

[34] Schulz KF, Altman DG, Moher D, Group C (2010). CONSORT 2010 statement: updated guidelines for reporting parallel group randomised trials. BMC Med.

[35] Gesundheitsförderung Schweiz (Health Promotion Switzerland). Friendly Work Space label. Health Promotion Switzerland. 2017 https://healthpromotion.ch/economy/instruments-and-services/friendly-work-space-label.html. Accessed 25.10.2017.

[36] Beer-Borst S, Haas K, Reinert R, Ryser C, für die Forschungsgruppe. Qualitätsstandards einer gesundheitsfördernden Gemeinschaftsgastronomie. Studienbericht zuhanden des Bundesamt für Gesundheit. Bern: Berner Fachhochschule, Fachbereich Gesundheit; 2010.

[37] Beer-Borst S, Krause C, Suter B, Haas KH, Ryser C, für die Forschungsgruppe. Gesundheitsfördernde Gemeinschaftsgastronomie. Systemische Umsetzung und kontinuierliche Erfolgskontrolle der Qualitätsstandards. Studienbericht zuhanden des Bundesamt für Gesundheit und der SV Stiftung. Bern: Berner Fachhochschule, Fachbereich Gesundheit; 2013.

[38] Nutbeam D, Smith C, Catford J (1990). Evaluation in health education. A review of progress, possibilities, and problems. J Epidemiol Community Health.

[39] Kwak L, Kremers SP, van Baak MA, Brug J (2006). Participation rates in worksite-based intervention studies: health promotion context as a crucial quality criterion. Health Promot Int.

[40] Mooney JD, Frank J, Anderson AS (2013). Workplace dietary improvement initiatives ought not to be discouraged by modest returns from low-intensity interventions. Eur J Pub Health.

[41] Maes L, Van Cauwenberghe E, Van Lippevelde W, Spittaels H, De Pauw E, Oppert JM (2012). Effectiveness of workplace interventions in Europe promoting healthy eating: a systematic review. Eur J Pub Health.

[42] Donfrancesco C, Ippolito R, Lo Noce C, Palmieri L, Iacone R, Russo O (2013). Excess dietary sodium and inadequate potassium intake in Italy: results of the MINISAL study. Nutr Metab Cardiovasc Dis.

[43] Nöhammer E, Stummer H, Schusterschitz C (2014). Employee perceived barriers to participation in worksite health promotion. J Public Health.

[44] Brandes S, Stark W. Empowerment / Befähigung. In: Leitbegriffe der Gesundheitsförderung. Bundeszentrale für gesundheitliche Aufklärung (BzgA). 2016. http://www.leitbegriffe.bzga.de/alphabetisches-verzeichnis/empowerment-befaehigung/. Accessed 25.10.2017.

[45] Brandstetter S, Ruter J, Curbach J, Loss J (2015). A systematic review on empowerment for healthy nutrition in health promotion. Public Health Nutr.

[46] Sorensen K, Van den Broucke S, Fullam J, Doyle G, Pelikan J, Slonska Z (2012). Health literacy and public health: a systematic review and integration of definitions and models. BMC Public Health.

[47] Kickbusch I, Pelikan JM, Apfel F, Tsouros AD (2013). Editors. Health literacy. The solid facts.

[48] Federal Commission for Nutrition (FCN). Iodine supply in Switzerland: current status and recommendations. Expert report of the FCN. Zurich: Federal Office of Public Health; 2013.

[49] World Health Organization (WHO). Ottawa Charter for Health Promotion 1986 http://www.who.int/healthpromotion/conferences/previous/ottawa/en/. Accessed 25.10.2017.

[50] Rosenbrock R, Hartung S. Setting Approach. In: Guiding concepts of Health Promotion and Disease Prevention. Bundeszentrale für gesundheitliche Aufklärung (BzgA). 2015. http://www.concepts-health-promotion.bzga.de/alphabetical-index/setting-approach-living-environment-approach/. Accessed 21.06.2017.

[51] Beer-Borst S, Haas K, Schader Ö, Siegenthaler S, Reinert R, Mühlemann P (2009). Nutritional quality in communal catering: a public health issue. Report on behalf of the Federal Office of Public Health and Bern University of applied sciences.

[52] Pathman D, Konrad T, Freed G, Freeman V, Koch G (1996). The awareness-to-adherence model of the steps to clinical guideline compliance: the case of pediatric vaccine recommendations. Med Care.

[53] Gilling SJ, Taylor EA, Kane K, Taylor JZ (2001). Successful hazard analysis critical control point implementation in the United Kingdom: understanding the barriers through the use of a behavioral adherence model. J Food Prot.

[54] Azanza MPV, Zamora-Luna MBV (2005). Barriers of HACCP team members to guideline adherence. Food Control.

[55] Forschungsgruppe Good Practice-Gemeinschaftsgastronomie, editor. Schweizer Qualitätsstandards für eine gesundheitsfördernde Gemeinschaftsgastronomie (Swiss quality standards for health-promoting communal catering). 2nd ed. Bern: Berner Fachhochschule, Fachbereich Gesundheit; 2015.

[56] Siegenthaler S, Beer-Borst S. Gesundheitsförderndes Verpflegungsangebot in der Gemeinschaftsgastronomie. Handbuch zur Durchführung einer Fachbegleitung. 2017. https://boris.unibe.ch/105567/. Accessed 25.10.2017.

[57] Krause C, Sommerhalder K, Beer-Borst S, Abel T. Just a subtle difference? Findings from a systematic review on definitions of nutrition literacy and food literacy. Health Promot Int. 2016; 10.1093/heapro/daw084. 10.1093/heapro/daw084 PMC6005107 27803197

[58] Nutbeam D (2008). The evolving concept of health literacy. Soc Sci Med.

[59] Nutbeam D (2000). Health literacy as a public health goal: a challenge for contemporary health education and communication strategies into the 21st century. Health Promot Int.

[60] Schwarzer R (2008). Modeling health behavior change: how to predict and modify the adoption and maintenance of health behaviors. Appl Psychol.

[61] Schwarzer R. Psychologie des Gesundheitsverhaltens. Einführung in die Gesundheitspsychologie. 3rd ed. Göttingen: Hogrefe; 2004.

[62] Hughes SL, Seymour RB, Campbell RT, Shaw JW, Fabiyi C, Sokas R (2011). Comparison of two health-promotion programs for older workers. Am J Public Health.

[63] Aneni EC, Roberson LL, Maziak W, Agatston AS, Feldman T, Rouseff M (2014). A systematic review of internet-based worksite wellness approaches for cardiovascular disease risk management: outcomes, challenges & opportunities. PLoS One.

[64] Jent S, Eisenblätter J. Förderung einer ausgewogenen, im Salz angepassten Ernährung im betrieblichen Umfeld. Manual zur Durchführung einer Ernährungsschulung. 2017. https://boris.unibe.ch/105569/. Accessed 25.10.2017.

[65] Forschungsgruppe Good Practice-Gemeinschaftsgastronomie. Checklisten zu den Qualitätsstandards für die Selbsteinschätzung der Betriebe als Word-Formulare zum Ausfüllen (Self-evaluation checklists). 2010. https://www.blv.admin.ch/blv/de/home/lebensmittel-und-ernaehrung/ernaehrung/massnahmen-ernaehrungsstrategie/gemeinschaftsgastronomie.html. Accessed 25.10.2017.

[66] Beer-Borst S. Questionnaires applied in the project „Healthful & Tasty: Sure!“ NRP69 salt consumption. 2017. https://boris.unibe.ch/106460/. Accessed 19.10.2017.

[67] Sorensen K, Pelikan JM, Rothlin F, Ganahl K, Slonska Z, Doyle G (2015). Health literacy in Europe: comparative results of the European health literacy survey (HLS-EU). Eur J Pub Health.

[68] Röthlin F, Pelikan JM, Ganahl K. Die Gesundheitskompetenz der 15-jährigen Jugendlichen in Österreich. Abschlussbericht der österreichischen Gesundheitskompetenz Jugendstudie im Auftrag des Hauptverbands der österreichischen Sozialversicherungsträger (HVSV). Wien; 2013.

[69] HLS-EU Consortium. Comparative Report of Health Literacy in Eight EU Member States. The European Health Literacy Survey HLS-EU. 2012. https://www.healthliteracyeurope.net/hls-eu. Accessed 28 Mar 2018.

[70] Krause C, Sommerhalder K, Beer-Borst S (2016). Nutrition-specific health literacy: development and testing of a multi-dimensional questionnaire. ErnahrungsUmschau.

[71] Grea Krause C, Beer-Borst S, Sommerhalder K, Hayoz S, Abel TA (2017). Short food literacy questionnaire (SFLQ) for adults: findings from a Swiss validation study. Appetite.

[72] World Health Organization/Pan American health organization regional expert Group for Cardiovascular Disease Prevention through population-wide dietary salt reduction. Sub-group for research and surveillance. Protocol for population level sodium determination in 24-hour urine samples. Geneva: WHO/PAHO; May 2010.

[73] Huang L, Crino M, Wu JH, Woodward M, Barzi F, Land MA (2016). Mean population salt intake estimated from 24-h urine samples and spot urine samples: a systematic review and meta-analysis. Int J Epidemiol.

[74] Beer-Borst S, Krause C, Siegenthaler S. Food Record Checklist V2.0 dated 04.12.2014/03.11.2016. Project "Healthful & Tasty: Sure!" NRP69 salt consumption. 2017. https://boris.unibe.ch/106666. Accessed 25.10.2017.

[75] Federal Food Safety and Veterinary Office (FSVO). Swiss Food Composition Database V5.0. 2013. http://www.naehrwertdaten.ch/. Accessed 25.10.2017.

[76] Mann SJ, Gerber LM (2010). Estimation of 24-hour sodium excretion from spot urine samples. J Clin Hypertens (Greenwich).

[77] Haldimann M, Zimmerli B, Als C, Gerber H (1998). Direct determination of urinary iodine by inductively coupled plasma mass spectrometry using isotope dilution with iodine-129. Clin Chem.

[78] Adriaens AG, Kelly WR, Adams FC (1993). Propagation of uncertainties in isotope-dilution mass-spectrometry using pulse counting detection. Anal Chem.

[79] Bates CJ, Thurnham DI, Bingham SA, Margetts BM, Nelson M, Margetts BM, Nelson M (1997). Biochemical markers of nutrient intake. Design concepts in nutritional epidemiology.

[80] Nath SK, Moinier B, Thuillier F, Rongier M, Desjeux JF (1992). Urinary excretion of iodide and fluoride from supplemented food grade salt. Int J Vitamin Nutr Res.

[81] IOM (Institute of Medicine), Food and Nutrition Board. Dietary reference intakes for vitamin A, vitamin K, arsenic, boron, chromium, copper, iodine, iron, manganese, molybdenum, nickel, silicon, vanadium, and zinc. Washington, DC: National Academy of Sciences; 2001.

[82] Forni Ogna V, Ogna A, Vuistiner P, Pruijm M, Ponte B, Ackermann D (2015). New anthropometry-based age- and sex-specific reference values for urinary 24-hour creatinine excretion based on the adult Swiss population. BMC Med.

[83] Beer-Borst S, Costanza MC, Pechère-Bertschi A, Wolff H, Burnier M, Morabia A. Calibration of Geneva adult population dietary sodium (salt) intake estimates from food frequency questionnaire (FFQ): validation study comparing FFQ versus 24-hour urinary measurements of sodium and potassium. Report on behalf of the Federal Office of Public Health. Geneva: Geneva University Hospital; 2006.

[84] Mancia G, Fagard R, Narkiewicz K, Redon J, Zanchetti A, Bohm M (2013). 2013 ESH/ESC guidelines for the management of arterial hypertension: the task force for the management of arterial hypertension of the European Society of Hypertension (ESH) and of the European Society of Cardiology (ESC). J Hypertens.

[85] Sebo P, Beer-Borst S, Haller DM, Bovier PA (2008). Reliability of doctors' anthropometric measurements to detect obesity. Prev Med.

[86] Wolff H, Delhumeau C, Beer-Borst S, Golay A, Costanza MC, Morabia A (2006). Converging prevalences of obesity across educational groups in Switzerland. Obesity (Silver Spring).

[87] Whigham LD, Schoeller DA, Johnson LK, Atkinson RL (2013). Effect of clothing weight on body weight. Int J Obes.

[88] World Health Organization (WHO). Obesity: preventing and managing the global epidemic. Report of a WHO consultation on obesity. Geneva: WHO; 2000. 11234459

[89] Ashwell M, Gunn P, Gibson S (2012). Waist-to-height ratio is a better screening tool than waist circumference and BMI for adult cardiometabolic risk factors: systematic review and meta-analysis. Obes Rev.

[90] Harris PA, Taylor R, Thielke R, Payne J, Gonzalez N, Conde JG (2009). Research electronic data capture (REDCap): a metadata-driven methodology and workflow process for providing translational research informatics support. J Biomed Inform.

[91] Luta X, Hayoz S, Gréa Krause C, Sommerhalder K, Roos E, Strazzullo P (2018). The relationship of health/food literacy and salt awareness to daily sodium and potassium intake among a workplace population in Switzerland. Nutr Metab Cardiovasc Dis.

[92] The R Foundation. The R Project for Statistical Computing. 2017. https://www.r-project.org/. Accessed 25.10.2017.

[93] Golden SD, Earp JA (2012). Social ecological approaches to individuals and their contexts: twenty years of health education & behavior health promotion interventions. Health Educ Behav.

[94] Chauhan BF, Jeyaraman M, Mann AS, Lys J, Skidmore B, Sibley KM (2017). Behavior change interventions and policies influencing primary healthcare professionals' practice-an overview of reviews. Implement Sci.

[95] Geaney F, Kelly C, Di Marrazzo JS, Harrington JM, Fitzgerald AP, Greiner BA (2016). The effect of complex workplace dietary interventions on employees' dietary intakes, nutrition knowledge and health status: a cluster controlled trial. Prev Med.

[96] Chu C, Breucker G, Harris N, Stitzel A, Gan XF, Gu XQ (2000). Health-promoting workplaces - international settings development. Health Promot Int.

[97] Ni Mhurchu C, Aston LM, Jebb SA (2010). Effects of worksite health promotion interventions on employee diets: a systematic review. BMC Public Health.

[98] Elliott P, Brown I. Sodium intakes around the world. Background document prepared for the forum and technical meeting on reducing salt intake in populations (Paris 5-7th October 2006). Geneva: World Health Organization; 2007.

[99] Konig F, Andersson M, Hotz K, Aeberli I, Zimmermann MB (2011). Ten repeat collections for urinary iodine from spot samples or 24-hour samples are needed to reliably estimate individual iodine status in women. J Nutr.

[100] Elwood JM (2007). Critical appraisal of epidemiological studies and clinical trials. Oxford - New York.

[101] Saturni S, Bellini F, Braido F, Paggiaro P, Sanduzzi A, Scichilone N (2014). Randomized controlled trials and real life studies. Approaches and methodologies: a clinical point of view. Pulm Pharmacol Ther.

[102] Odgaard-Jensen J, Vist GE, Timmer A, Kunz R, Akl EA, Schunemann H, et al. Randomisation to protect against selection bias in healthcare trials. Cochrane Database Syst Rev. 2011(4):MR000012. 10.1002/14651858.MR000012.pub3 PMC7150228 21491415

[103] Herson J, Carter SK (1986). Calibrated phase II clinical trials in oncology. Stat Med.

[104] Glasgow RE, Vogt TM, Boles SM (1999). Evaluating the public health impact of health promotion interventions: the RE-AIM framework. Am J Public Health.

